# Myocardial Extracellular Matrix Volume has independent prognostic value in patients with non-ischemic cardiomyopathy

**DOI:** 10.1186/1532-429X-18-S1-P226

**Published:** 2016-01-27

**Authors:** Tomas S Vita, Ethan Rowin, Siddique Abbasi, Xiaohu Li, Hoshang Farhad, Tomas G Neilan, Eri Watanabe, François-Pierre Mongeon, Michael Givertz, Michael Steigner, Ron Blankstein, Marcelo DiCarli, Michael Jerosch-Herold, Raymond Y Kwong

**Affiliations:** grid.62560.370000000403788294Brigham and Womens Hospital, Boston, MA USA

## Background

Left ventricular ejection fraction (LVEF) is the most validated independent prognostic factor for mortality, ventricular arrhythmias and heart failure (HF) in non-ischemic dilated cardiomyopathy without etiology. However, mild or moderate reduction LVEF yield limited predictive value.

The amount of fibrosis, detected by late gadolinium enhancement (LGE) on CMR, has also been validated as an independent predictor factor.

Conventional imaging techniques cannot robustly quantify the full spectrum of extracellular cardiac matrix volume (ECV) expansion. ECV expansion often may not be evident on LGE CMR or other modalities.

Quantifying ECV may ultimately provide independent prognostic value to improve care through targeted treatment.

The aim of this study was to determine the prognostic value of myocardial ECV expansion in patients with non-ischemic cardiomyopathy.

## Methods

Patients clinically referred for cardiac MRI evaluation between December 2008 and January 2014 with non-ischemic cardiomyopathy with LVEF<60% were included in the study cohort. Primary endpoints included death and heart failure (HF) admissions. Elevated ECV was based on 2SD above normal volunteers sampling, at a cutoff of 34%. T1 measurements were performed with a cine Look-Locker sequence non-slice-selective inversion pulse, followed by segmented gradient-echo acquisition for 17 cardiac phases after inversion, spread over 2 cardiac cycles.

## Results

Three hundred and fifty nine patients were included. The mean age was 50 +/- 16 years; 60% were male; LV EF was 46 +/- 10%; LV end diastole and end systole volume index (mL/m2) were 98+ 34 and 56+34, respectively. Mean LV mass was 60+/-21 g. At a median follow-up of 2.5 yrs (IQR 2.5 yrs), 22 deaths and 24 HF admissions resulted. LGE was positive in 32% of patients. Depressed LV EF under 30% was present in 15% of the population. Twenty five percent of patients had an ECV mean over 34%. Adjusted for LV EF <30% and LGE presence, elevated ECV showed a significant association with death (p=0.009; HR=3.83;CI=1.39-10.51).

## Conclusions

1. ECV provides prognostic value for mortality of patients with non-ischemic CMP, incremental to LVEF and presence of myocardial scar by CMR.

2. Adjusted to LVEF of <30% and LGE presence, ECV mean > 34% portends to a near 4-fold increased risk for death.

**Figure 1:**
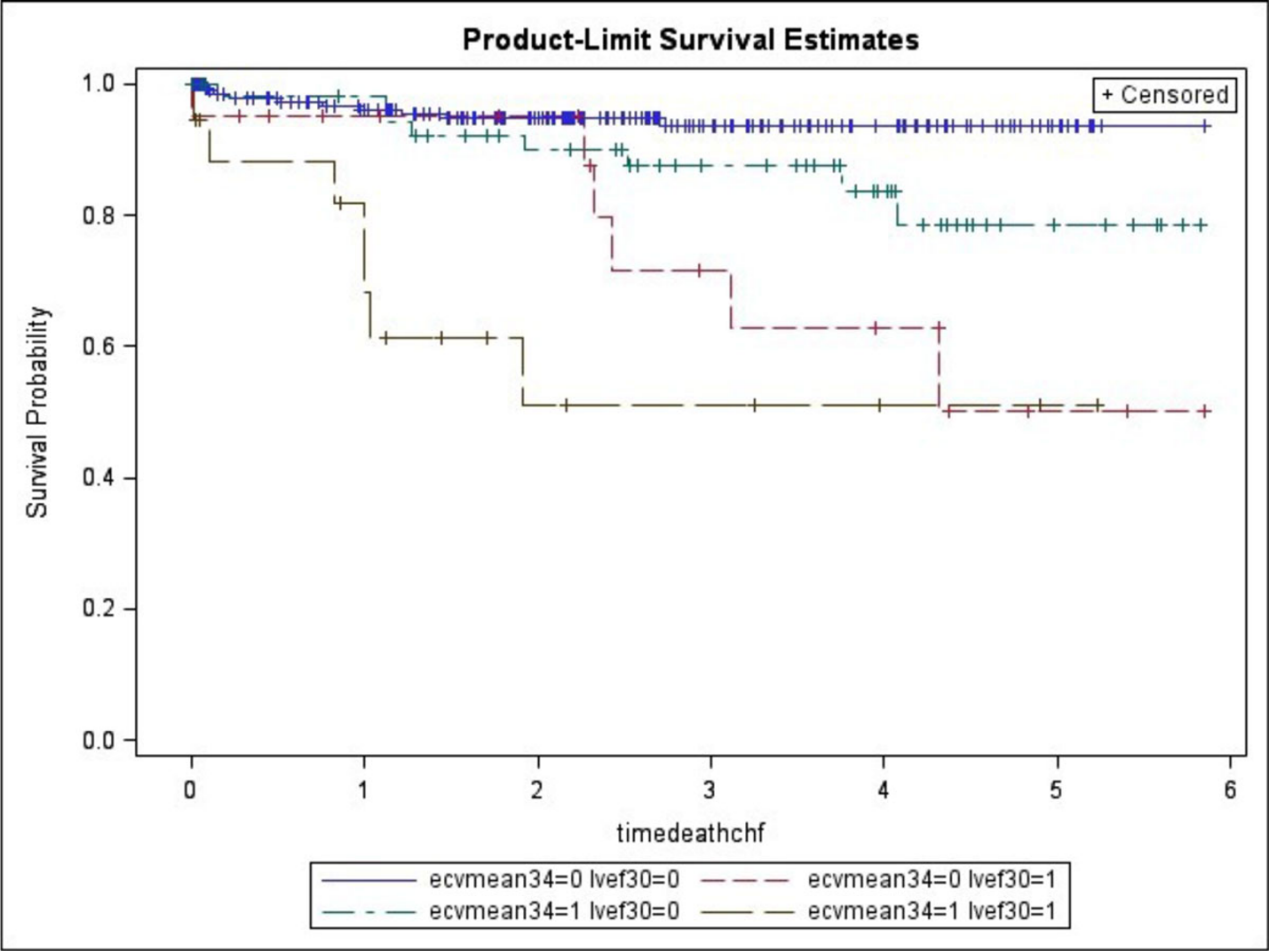
**Plots of Kaplan-Meier product limit estimates of survival based on ECV and LVEF**.

